# The Vegetable Kingdom; or the Structure, Classification, and Uses of Plants, Illustrated upon the Natural System

**Published:** 1846-10

**Authors:** 


ART. IV.—*The Vegetable Kingdom; or the Structure, Classification, and Uses of Plants, illustrated upon the Natural System.* By JOHN LINDLEY, PH.D., F.R.S., F.L.S., Professor of Botany in the University of London, and in the Royal Institution of Great Britain. With upwards of Five Hundred Illustrations.—*London,* 1846. 8vo, pp. 908.

THIS work, which we may really call a splendid one, may be considered as a third edition of Dr. Lindley's Introduction to the Natural System of Botany, of which the first was published in 1830. At that period, the system of De Candolle was in general estimation amongst those botanists who had the wisdom to discern the absurdity of adhering to the Linnæan or artificial system, long after its author would have disowned it. The progress of science, however, showed many imperfections and inconsistencies in De Candolle's arrangement, particularly as regarded the want of some intermediate subdivisions between the few primary groups of Phanerogamia and the hundreds of natural orders which were even then recognized. And in his second edition, published in 1836, Dr. Lindley attempted to carry out a plan, which had been proposed by some continental writers, of grouping these orders into alliances. The ten years which have elapsed since that period, have served to confirm the general truth of the views which he then propounded, whilst they have rendered necessary many modifications as to details. The new materials continually presenting themselves to the botanical architect, for which a place *must* be found in his edifice, require continual shiftings and alterations in his minor arrangements, although the principles of construction may remain the same; and consequently it is as absurd to criticise him for departures from his former plans, as it would be to find fault with a chemist or a physiologist for making discoveries which upset or modify some previous system. "In fact," as Dr. Lindley justly remarks, "there is no such thing as stability in these matters. Consistency is but another name for obstinacy. All things are undergoing incessant change. Every science is in a state of progression, and of all others the sciences of observation the most so." And with most commendable humility he proceeds: "The author's object, and he thinks he may say that of every one else who has turned his attention to the question of late, has not been to establish a system of his own, which shall be immutable, but to contribute to the extent of his ability towards that end. . . . . All that we can do is to throw our pebbles upon the heap, which shall hereafter, when they have sufficiently accumulated, become the landmark of systematic botany."

The following outline of the contents of this volume will indicate the vast amount of matter contained in it, and will give some idea of the labour which must have been bestowed upon its composition:

"Its object is to give a concise view of the state of systematic botany at the present day, to show the real or supposed relation of one group of plants to another, to explain their geographical distribution, and to point out the various uses to which the species are applied in different countries. The names of all known genera, with their synonyms, are given under each natural order, the numbers of genera and species are in every case computed from what seems the best authority, and complete indices of the multitude of names embodied in the work are added, so as to enable a botanist to know immediately under what natural order a given genus is stationed, or what are the uses to which any species has been applied. Finally, the work is copiously illustrated by wood and glyphographic cuts; and, for the convenience of students, an artificial analysis of the system is placed at the end." (Preface, p. xii.)

It would be quite beyond our province to attempt any detailed criticism on such a masterly compendium of botanical science as the one now before us. And we shall content ourselves with recording our conviction, that no work of anything like equal value exists either in our own language or in any other; that it is consequently indispensable to every student who desires to make himself acquainted with the present aspect of botanical science, besides serving as a most valuable work of reference to those who desire only occasional information as to the characters, properties, and uses of the various species of plants (to the acquirement of which the copious indices give every facility); and that Dr. Lindley well deserves the gratitude of all botanists for the zeal and perseverance which could alone have carried him through a work of such immense labour.

We cannot put aside the book, however, without remarking that, in our opinion, Dr. Lindley's attempt to Anglicize scientific names may be very well spared, and is in fact injurious. In a *popular* treatise, it is very right to endeavour to ingraft science upon facts of familiar knowledge, and to show that the many-syllabled Euphorbiaceæ and Ranunculaceæ are nothing but Spurges and Crowfoots, or plants allied to them. But in a scientific work, intended for foreigners as well as natives, the common language of science ought, we think, to be rigorously maintained; and we cannot see the advantage, either in euphony or appearance, of Hydrocharads over Hydrocharidaceæ, Bromelworts over Bromeliaceæ, Pontederads over Pontederaceæ, or Kadsurads over Schizandraceæ.

